# Moving the Needle Cautiously in Targeting One of the Most Often Acquired Receptor Tyrosine Fusion (*RET* Fusion) Resistance Mechanisms to EGFR Tyrosine Kinase Inhibitors

**DOI:** 10.1016/j.jtocrr.2022.100358

**Published:** 2022-08-17

**Authors:** Shannon S. Zhang, Sai-Hong Ignatius Ou

**Affiliations:** a Department of Medicine, University of California, Irvine School of Medicine, Orange, California; b Chao Family Comprehensive Cancer Center, Orange, California

Pralsetinib and selpercatinib are two selective *RET* tyrosine kinase inhibitors (TKIs) approved for the treatment of *RET* fusion-positive NSCLC, *RET* fusion-positive thyroid cancer, and medullary thyroid cancer harboring *RET* mutations in 2020,^1^^,^^2^ barely eight years after the discovery and report of *RET* fusions in NSCLC in 2012.^3^, ^4^, ^5^, ^6^ Approvals of these two *RET* TKIs implied a recognition by the U.S. Food and Drug Administration that *RET* fusions are an unique and validated targetable driver mutation in oncology.

De novo activating *EGFR* mutations occur in approximately 50% of NSCLC in Asia.^7^ Resistance to *EGFR* TKIs invariably occurs, and receptor tyrosine kinase (RTK) fusion is an uncommon but increasingly recognized acquired resistance mechanism. Acquired RTK fusions occurring in the setting of all generations of *EGFR* TKIs and *RET* fusions seem to be the most common RTK fusion reported in the literature.^8^ Although per the Food and Drug Administration approval summary, *RET* fusions occurred in 1% to 2% of NSCLC, a relatively uncommon mutation (1.2), the commercial availability of these two *RET* TKIs allows the rare occasional off-label use of these TKIs to target *RET* fusions as a secondary acquired resistance mechanisms especially to *EGFR* TKIs. In fact, as proof of principle, the combination of pralsetinib with osimertinib has been reported in two patients with *EGFR+* NSCLC to overcome acquired RET fusion (*CCDC6-RET* and *NCOA4-RET*, respectively) as resistance to *EGFR* TKIs.^9^ In the June, 2022 issue of JTO Clinical and Research Reports, Zhao et al.^10^ reported a similar case report of *CCDC6-RET* fusion arising as an acquired resistance to dacomitinib as the most immediate *EGFR* TKI in an *EGFR+* L858R/V834L. Missing in their report is the allele frequency of the *EGFR* L858R and V834L mutations to let the readers know whether V834L is in the same tumor clone as L858R or is a minor separate clone.

Nevertheless, Zhao et al.^10^ reported that this combination was able to symptomatically treat leptomeningeal carcinomatosis. Without cytology from lumbar puncture to evaluate the molecular profile of the tumor cells in the cerebrospinal fluid (CSF), however, it is impossible to pinpoint whether switching to osimertinib alone or the combination with pralsetinib led to the symptomatic improvement from the leptomeningeal carcinomatosis.

Importantly, the CSF and plasma concentrations of both osimertinib and pralsetinib were measured at a steady state 4 months after commencing the combination therapy. As the authors pointed, a simultaneous CSF and plasma pharmacokinetics of pralsetinib monotherapy has not been reported but was performed by the authors at the full approved dose of 400 mg once daily of pralsetinib in combination with osimertinib 80 mg daily. From the package insert, the unbound plasma fraction of pralsetinib is 2.9% (97.1% protein binding),^11^ and the calculated unbound CSF-to-plasma ratio of the patient is 0.27. Although the CSF and plasma pharmacokinetic levels drawn were in combination with osimertinib, important missing information is whether osimertinib had any effect on the pralsetinib dose level and vice versa as Zhao et al.^10^ have discussed in their brief report.

An important consideration is our experience that the combination of osimertinib and pralsetinib required frequent and detailed clinical follow up, especially in the early months of this combination. Both osimertinib and pralsetinib had overlapping toxicities, such as leukopenia, thrombocytopenia, liver enzyme elevation, and particularly and most importantly pneumonitis. The incidence of pneumonitis (all grade) from pralsetinib is 10% (with 2.7% grades 3–4, 0.5% fatal) listed in the current U.S. package insert,^11^ whereas pneumonitis occurred in 3.3% (0.5% fatal) of patients treated with osimertinib per the U.S. package insert.^12^ In our previous report, both patients started at osimertinib 80 mg daily and pralsetinib 200 mg daily with titration of pralsetinib at a 100 mg dose increment every 2 weeks to a goal of 400 mg once daily (the approved monotherapy dose). One patient was eventually treated with osimertinib (80 mg daily) plus pralsetinib (300 mg daily). Another patient (our patient) who was eventually dose escalated to osimertinib (80 mg daily) plus pralsetinib (400 mg daily at 100 mg increment starting at 200 mg once daily) had to be dose reduced back to pralsetinib 200 mg once daily and osimertinib (80 mg once daily) due to leukopenia and grade 1 pneumonitis but who enjoyed a durable response of more than 2 years. Thus, we do not recommend immediately starting both osimertinib and pralsetinib at a full dose as Zhao et al.^10^ have done.

Ideally, it will be important to detect the presence and monitor the disappearance of *CCDC6-RET* fusion variants in the plasma. Another important future learning experience will be to report the eventual resistance mechanism to this combination of osimertinib and pralsetinib combination. Finally, the combination of selpercatinib with EGFR TKI should also be investigated. Selpercatinib has been combined successfully with crizotinib^13^ and capmatinib.^14^ To extend our argument, RTK fusions (*EML4-ALK*, *CCDC6-RET*, *FGFR3-TACC3*) have been shown to constitute part of the spectrum of the resistance to covalent KRAS G12C inhibitors.^15^ Thus we anticipate more case reports on combination with RTK inhibitors and inhibitors of other actionable driver mutation inhibitors such as RET TKI + KRAS G12C inhibitor will be forthcoming in the future. However for these novel approaches to overcome acquired resistance to be attempted, the ability to comprehensively assay for acquired resistance mechanism to TKIs or KRAS G12C inhibitors first have to be widely adopted.

In conclusion, this case report provided more proof of principle of targeting acquired RTK fusions together with EGFR TKI to combat resistance to EGFR TKI and may expand our horizon to target resistance to covalent KRAS G12C inhibitors with the caveat to take into consideration overlapping toxicities regardless of whether the combination treatment is under a protocol or off-label use. There is an urgent need for developing multicohort combination protocols targeting emergent acquired RTK fusions as a resistance mechanism to targeted therapies to investigate tolerability and efficacy, for example, pralsetinib or selpercatinib in combination with osimertinib for *RET* fusion mediated resistance to EGFR TKIs or pralsetinib or selpercatinib in combination with sotorasib (or other KRAS G12C inhibitors) for acquired *RET* fusion mediated resistance to KRAS G12C inhibitors.

## CRediT Authorship Contribution Statement

**Shannon S. Zhang:** Conceptualization, Data curation, Writing - original draft, Writing - review & editing.

**Sai-Hong Ignatius Ou:** Conceptualization, Data curation, Supervision, Writing - original draft, Writing - review & editing.
